# Inhibition of JNK increases the sensitivity of hepatocellular carcinoma cells to lysosomotropic drugs via LAMP2A destabilization

**DOI:** 10.1038/s41420-021-00408-0

**Published:** 2021-02-08

**Authors:** Enrico Desideri, Maria Rosa Ciriolo

**Affiliations:** 1 Department of Biology, University of Rome “Tor Vergata”, Via della Ricerca Scientifica 1, 00133 Rome, Italy; 2 IRCCS San Raffaele Pisana, Via della Pisana 235, 00163 Rome, Italy

**Keywords:** Chemotherapy, Hepatocellular carcinoma, Lysosomes

## Abstract

Alteration of lysosomal homeostasis is common in cancer cells, which often feature an enlarged and peripheral distributed lysosomal compartment and the overexpression of cathepsins. These alterations accelerate the production of building blocks for the de novo synthesis of macromolecules and contribute to the degradation of the extracellular matrix, thus contributing to tumor growth and invasion. At the same time, they make lysosomes more fragile and more prone to lysosomal membrane permeabilization, a condition that can cause the release of proteases into the cytosol and the activation of cell death. Therefore, lysosomes represent a weak spot of cancer cells that can be targeted for therapeutic purposes. Here, we identify a novel role of the kinase JNK as keeper of lysosomal stability in hepatocellular carcinoma cells. JNK inhibition reduces the stability of LAMP2A, a lysosomal membrane protein responsible for the stability of the lysosomal membrane, promoting its degradation by the proteasome. LAMP2A decrease enhances the lysosomal damage induced by lysosomotropic agents, ultimately leading to cell death. The effect is cancer-specific, as JNK inhibition does not decrease LAMP2A in non-tumoral liver cells and does not alter their sensitivity to lysosomotropic drugs. Our finding on the new role of JNK as cancer-specific keeper of lysosomal homeostasis lays the ground for future evaluation of the efficacy of the combination of JNK inhibition and lysosomotropic agents as a potential therapeutic strategy in hepatocellular carcinoma.

## Introduction

Lysosomes are membrane-bound acidic organelles whose function goes beyond the degradation of macromolecules deriving from the intracellular and extracellular space. In fact, they participate in many cellular functions, including nutrient sensing, metabolic signaling, plasma membrane repair, and secretion^1^. Vacuolar ATPases localized at the lysosomal membrane maintains the lysosomal lumen acid (pH around 4.5). The low pH promotes the activity of lysosomal hydrolases, which degrade proteins, lipids, sugars, and nucleic acids into their building blocks necessary for the de novo synthesis of macromolecules^2^. The lysosomal membrane is protected from self-digestion by proteases, such as cathepsins, by a thick layer of glycocalyx made of sugar residues present in the luminal portion of membrane proteins^3^. Loss of lysosomal integrity is hazardous for the cells because it may lead to lysosomal membrane permeabilization (LMP), the release of cathepsins in the cytosol and activation of cell death mechanisms^4^. The most abundant lysosomal membrane proteins are the lysosomal-associated membrane proteins 1 (LAMP1) and 2 (LAMP2). Both proteins contain a highly glycosylated luminal region, a single-spanning transmembrane domain and a small C-terminal cytosolic tail. LAMPs are essential to maintain lysosomal integrity and functionality and are also involved in other cellular functions, such as autophagy^3^.

*LAMP2* gene encodes for three proteins, LAMP2A, LAMP2B, and LAMP2C, through an event of alternative splicing involving the exon 9 (in human)^5^. The three isoforms share an identical luminal portion and have a different transmembrane and cytosolic region. Each isoform is expressed at variable levels in the different tissues and plays distinct roles. The most studied isoform, LAMP2A, is highly expressed in the liver, kidney, and placenta^6^. LAMP2A expression changes in response to stress conditions, such as nutrient deprivation and oxidative stress, and is the limiting factor of chaperone-mediated autophagy^7^.

Alteration of lysosomal structure and activity is a feature of tumor cells, which often show an enlarged lysosomal compartment, characterized by a more peripheral distribution than normal cells and higher expression and activity of cathepsins^8^. Lysosomal alterations support tumor growth in different ways. For example, overexpression of cathepsins accelerates the degradation of cellular material, increasing the availability of building blocks required for tumor growth^8^. Lysosomes can also sequester chemotherapeutic agents, preventing them from reaching their targets, thus contributing to chemoresistance^9,10^. Additionally, lysosomes can fuse with the plasma membrane and release cathepsins outside the cells, triggering the degradation of the extracellular matrix and favoring tumor invasion and angiogenesis^11^. However, the increased lysosomal activity makes lysosomes of cancer cells more fragile and more prone to LMP than their normal counterpart, making them good therapeutic targets^12^. Given the pivotal role of LAMP2 in lysosomal stability, targeting LAMP2 could be a strategy to destabilize lysosomes of cancer cells and potentiate the efficacy of lysosomotropic agents, which accumulates into lysosomes and damage them, resulting in LMP and activation of cell death^13^. Importantly, lysosomal damage can activate several cell death mechanisms, including apoptosis, necrosis, and necroptosis, and can represent a way to circumvent the resistance to apoptosis typical of many cancer cells^14^. So far, the knowledge of the upstream regulators of LAMP2 is still partial, and only a few factors have been identified^15–17^. A recently identified regulator of LAMP2 is the mitogen-activated kinase (MAPK) p38, which phosphorylates and stabilizes LAMP2A^18^.

MAPKs are a family of serine/threonine kinases activated by several extracellular and intracellular stimuli like growth factors, cytokines, and a variety of cellular stresses^19^. MAPKs are activated by phosphorylation and in turn phosphorylate a plethora of cytosolic and nuclear targets, through which they regulate many cellular functions, including proliferation, cell death, and differentiation. The MAPK family includes, in addition to p38, the c-Jun N-terminal kinase (JNK), the extracellular signal-regulated kinase (ERK), and the extracellular-signal-regulated kinase 5 (ERK5)^20^. JNK and p38 are usually involved in stress response and are often referred to as stress-activated protein kinases (SAPKs)^21^. JNK exists in three isoforms, JNK1, JNK2, and JNK3, encoded by distinct genes, *MAPK8*, *MAPK9*, and *MAPK10* ^22^. JNK1 and JNK2 are ubiquitously expressed, while JNK3 expression is restricted to the brain and heart ^23^. Once activated by the MAPK kinase 4 and 7, JNK phosphorylates many downstream targets, which include the transcription factors c-Jun and the nuclear factor of activated T‐cell (NFAT)^24^. Interestingly, NFAT positively regulates LAMP2A expression in T-cells^17^, suggesting a possible role of JNK in the control of LAMP2A expression and, in turn, lysosomal homeostasis. To date, JNK was already shown to regulate autophagy positively, but whether it also has a role in lysosomal homeostasis is not known^25^. JNK pathway has been associated with hepatocellular carcinoma (HCC), the primary liver malignancy and a type of cancer with limited therapeutic options^26^. In HCC, JNK can play either tumor-suppressing or tumor-promoting functions, and it also contributes to chemoresistance^23^. In this work, we investigated the effect of JNK modulation on LAMP2A and lysosomal homeostasis in HCC cells. We show that inhibition of JNK reduces LAMP2A stability in HCC cells and potentiates the toxic effects of lysosomotropic agents. This effect is cancer-specific, as JNK does not influence LAMP2A expression in an immortalized liver cell line, revealing a difference between tumor and normal cells that could be exploited for therapeutic purposes.

## Results

### JNK controls LAMP2A stability

**Fig. 1:**
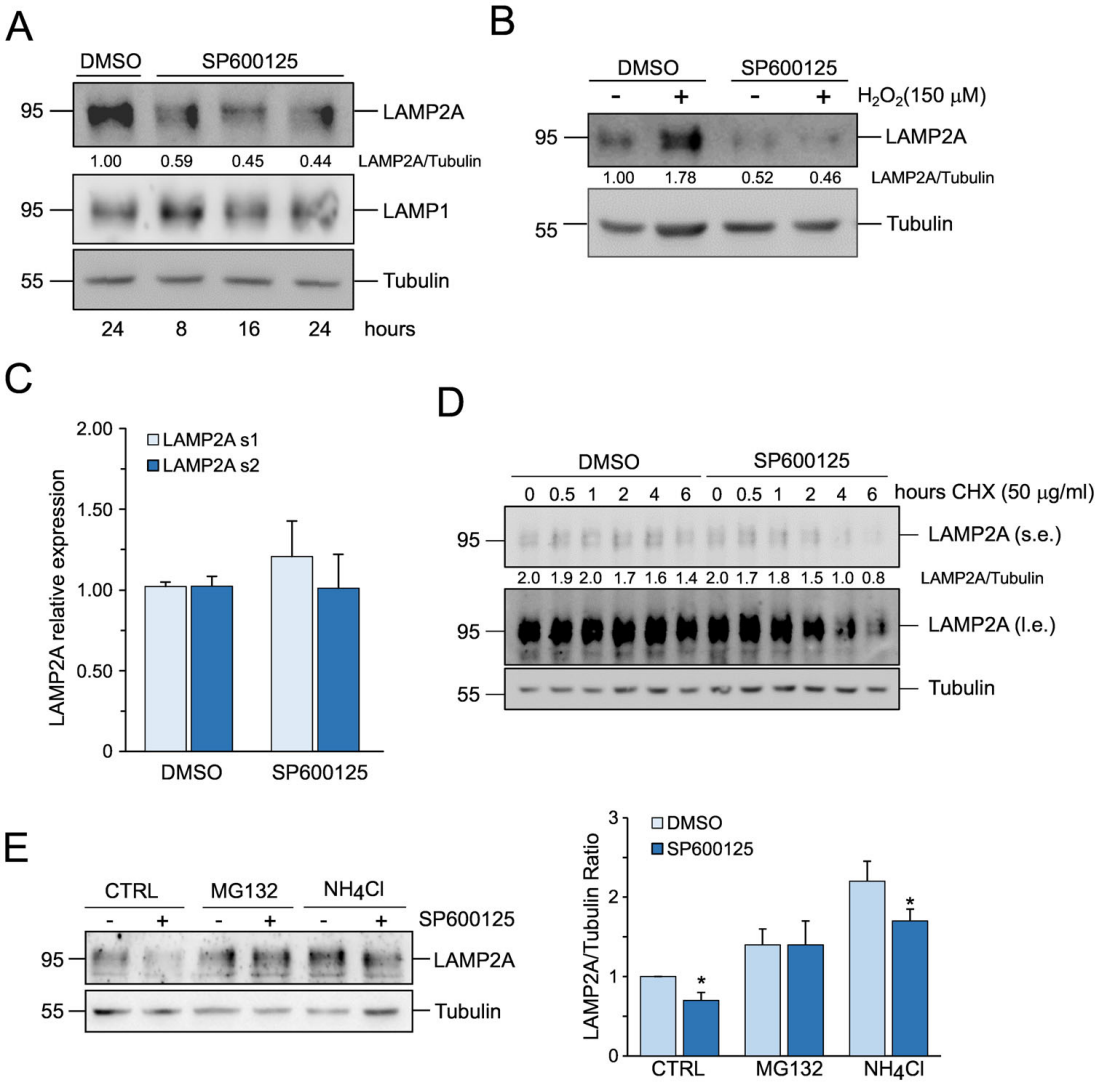
JNK regulates LAMP2A stability. **A** Western blot analysis of LAMP2A and LAMP1 expression in Hep3B cells treated with 10 μM SP600125 for the indicated times. **B** Western blot analysis of LAMP2A expression in Hep3B cells treated for 6 h with 150 μM H_2_O_2_ in the presence or absence of 10 μM SP600125. **C** Real-time qPCR analysis of LAMP2A mRNA expression in Hep3B cells treated for 24 h with 10 μM SP600125. Data are expressed as mean ± SEM of *n* = 3 independent experiments. Actin was used as a reference gene. **D** Western blot analysis of LAMP2A expression of Hep3B cells treated with 50 μg/ml CHX for the indicated time in the presence or absence of 10 μM SP600125. **E** (left panel) Western blot analysis of LAMP2A expression in Hep3B cells treated with 10 μM SP600125 for 16 h in the presence of either 5 μM MG132 or 20 mM NH_4_Cl. (right panel) Densitometric analysis of LAMP2A expression. Data are expressed as mean ± SEM of *n* = 3 independent experiments. Data were normalized to tubulin. *p < 0.05 vs. DMSO. For Western blots, tubulin was used as a loading control. Western blots are representative of *n* = 3 experiments showing similar results.

To check whether JNK manipulation influenced LAMP2A, we treated Hep3B cells with 10 μM of the JNK inhibitor SP600125 for 8, 16, and 24 h. Western blot analysis of LAMP2A showed that JNK inhibition decreased LAMP2A levels (Fig. 1A). On the contrary, LAMP1 is not influenced by JNK inhibition, indicating that the effect of JNK is specific for LAMP2A. Next, we pre-treated Hep3B cells with 10 μM SP600125 for 30 min and then treated them with 150 μM H_2_O_2_, which promoted the accumulation of LAMP2A, for an additional 6 h. Even under this condition, JNK inhibition completely blocked LAMP2A accumulation (Fig. 1B). Next, we investigated the causes of LAMP2A decrease upon JNK inhibition. The evidence that the transcription factor and JNK substrate NFAT regulates the expression of LAMP2A in T cells prompted us to test the hypothesis that JNK could regulate LAMP2A transcription. We performed a real-time qPCR analysis of LAMP2A mRNA in Hep3B cells treated for 24 h with 10 μM SP600125. Figure 1C clearly shows that JNK inhibition did not affect LAMP2A mRNA. Then, we tested whether JNK regulated LAMP2A protein stability by pre-treating Hep3B cells with 10 μM SP600125 for 30 min and then treating them with 50 μg/ml of the translation inhibitor cycloheximide (CHX) for an additional 0.5, 1, 2, 4, and 6 h. LAMP2A levels remained constant throughout the entire experimental time in control cells, whereas in the presence of the JNK inhibitor LAMP2A started to decrease from 2 h of CHX, indicating that JNK regulated the stability of LAMP2A (Fig. 1D). To identify the mechanism responsible for LAMP2A degradation when JNK was inhibited, we treated Hep3B cells for 16 h with either 5 μM of the proteasome inhibitor MG132 or 20 mM of the lysosomal inhibitor NH_4_Cl, in the presence or absence of 10 μM SP600125. Inhibition of the proteasome did not significantly raise LAMP2A in unstimulated cells, but it was able to prevent the decline of LAMP2A caused by JNK inhibition, whereas the inhibition of lysosomal activity failed, although it was able to raise basal LAMP2A levels (Fig. 1E). These results demonstrate that JNK preserved LAMP2A stability and prevented its degradation by the proteasome.

### JNK inhibition perturbs lysosomal homeostasis and potentiates the efficacy of lysosomotropic agents

**Fig. 2:**
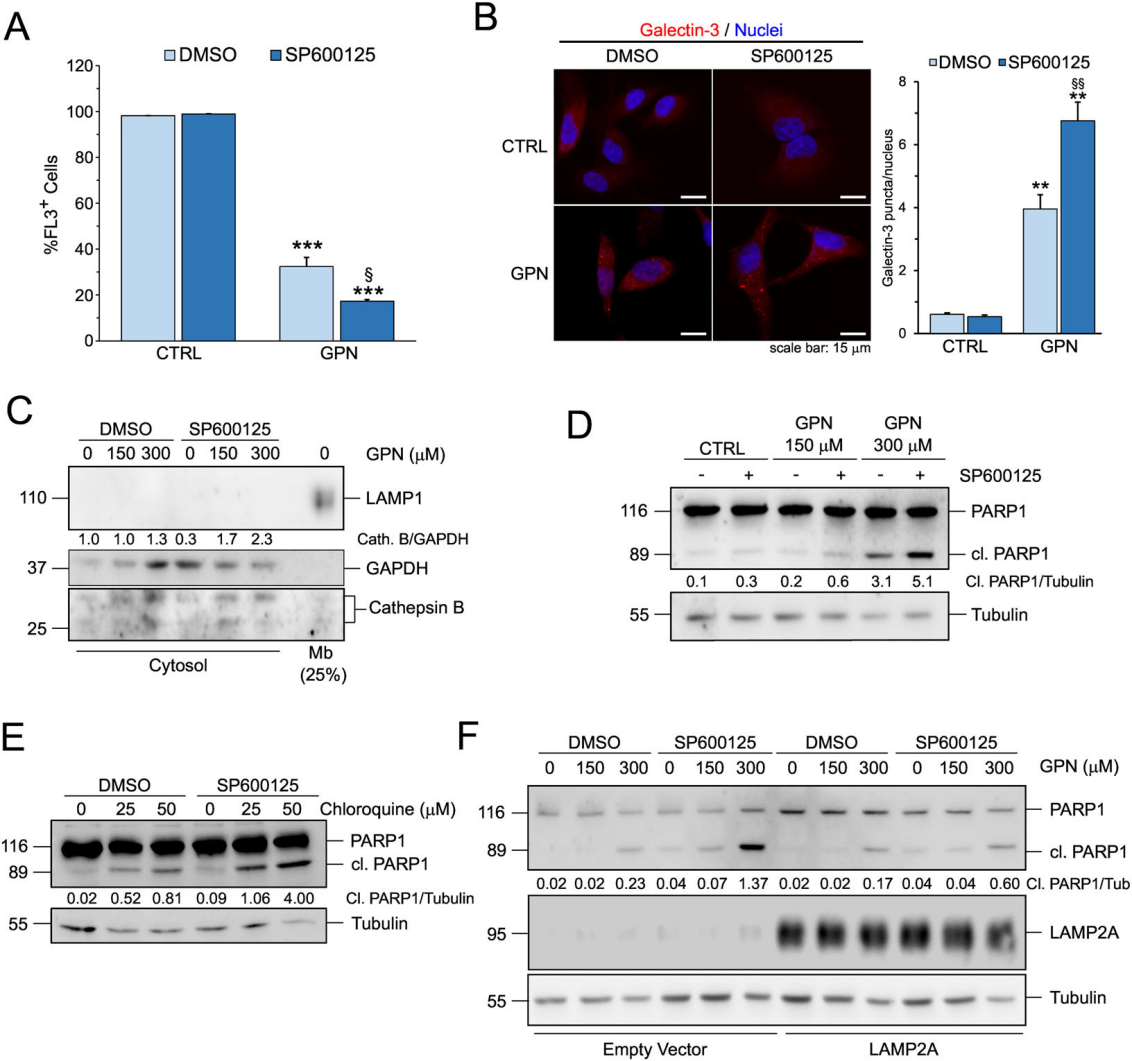
JNK inhibition impacts on lysosomal homeostasis. **A** Cytofluorometric analysis of intracellular acidification using acridine orange in Hep3B pre-treated for 6 h with 10 μM SP600125 and then treated with 300 μM GPN for an additional 2 h. Data are expressed as mean ± SEM of *n* = 3 independent experiments. ***p < 0.001 vs. CTRL; ^§^*p* < 0.05 vs. DMSO. **B** Fluorescence microscopy analysis of galectin-3 puncta in Hep3B pre-treated for 6 h with 10 μM SP600125 and then treated with 300 μM GPN for an additional 2 h. Data are expressed as mean ± SEM of *n* = 3 independent experiments. **p < 0.01 vs. CTRL; ^§§^*p* < 0.01 vs. DMSO. **C** Western blot analysis of the release of cathepsin-B in the cytosol in Hep3B pre-treated for 6 h with 10 μM SP600125 and then treated with GPN for an additional 2 h. LAMP1 and GAPDH were used as controls of fraction purity. **D** and **E** Western blot analysis of PARP1 in Hep3B treated for 24 h with the indicated concentration of the lysosomotropic agents GPN (**D**) and chloroquine (**E**) in the presence or absence of 10 μM SP600125. **F** Western blot analysis of PARP1 in Hep3B overexpressing LAMP2A and treated for 24 h with the indicated concentration of the lysosomotropic agents GPN in the presence or absence of 10 μM SP600125. Where applicable, tubulin was used as a loading control. Western blots are representative of *n* = 3 independent experiments showing similar results.

Given the prominent role of LAMP2A in maintaining lysosomal homeostasis, we evaluated the effect of JNK inhibition on the lysosomes. We treated Hep3B cells with the lysosome damaging agent glycyl-l-phenylalanine 2-naphthylamide (GPN), in the presence or absence of 10 μM SP600125. To evaluate lysosomal homeostasis we monitored cellular acidification, which greatly depends on the lysosomes, the presence of cathepsin B into the cytosol and the appearance of galectin-3 puncta, which are indicators of lysosomal damage^27^. Hep3B cells treated with 10 μM SP600125 did not show any alteration of lysosomal homeostasis (Fig. 2A–C). However, when used in combination with the lysosomal damaging agent GPN, JNK inhibition potentiated the effects of GPN, causing a more sustained drop in lysosomal acidification (Fig. 2A), an increase of galectin-3 puncta (Fig. 2B) and the release of cathepsin B into the cytosol at lower doses of GPN (Fig. 2C). These results clearly indicate that JNK inhibition makes lysosomes more fragile and susceptible to damage. Next, we tested whether the increased lysosomal fragility when JNK is inhibited could result in an increased cell death in cells challenged with lysosomotropic agents. For this reason, we treated Hep3B cells for 24 h with either GPN or chloroquine (CQ), in the presence or absence of 10μM SP600125. Results clearly showed that the combination of JNK inhibition and either GPN or CQ increased cell death, as demonstrated by the appearance of the cleaved fragment of the caspase 3 substrate poly [ADP-ribose] polymerase 1 (PARP1) (Fig. 2D and E). Finally, to confirm that the increased cell death could be attributed to the effect of JNK on LAMP2A, we transfected Hep3B cells either with an empty vector or with a plasmid expressing LAMP2A and treated them for 24 h with GPN, in the presence or absence of 10 μM SP600125. As shown in Fig. 2F, LAMP2A overexpression did not protect the cell by the effect of GPN alone, but it counteracted the synergistic effect of JNK inhibition. Altogether, these results indicated that JNK contributes to maintaining lysosomal homeostasis via LAMP2A.

### JNK regulation of LAMP2A is a cancer-specific phenomenon

**Fig. 3:**
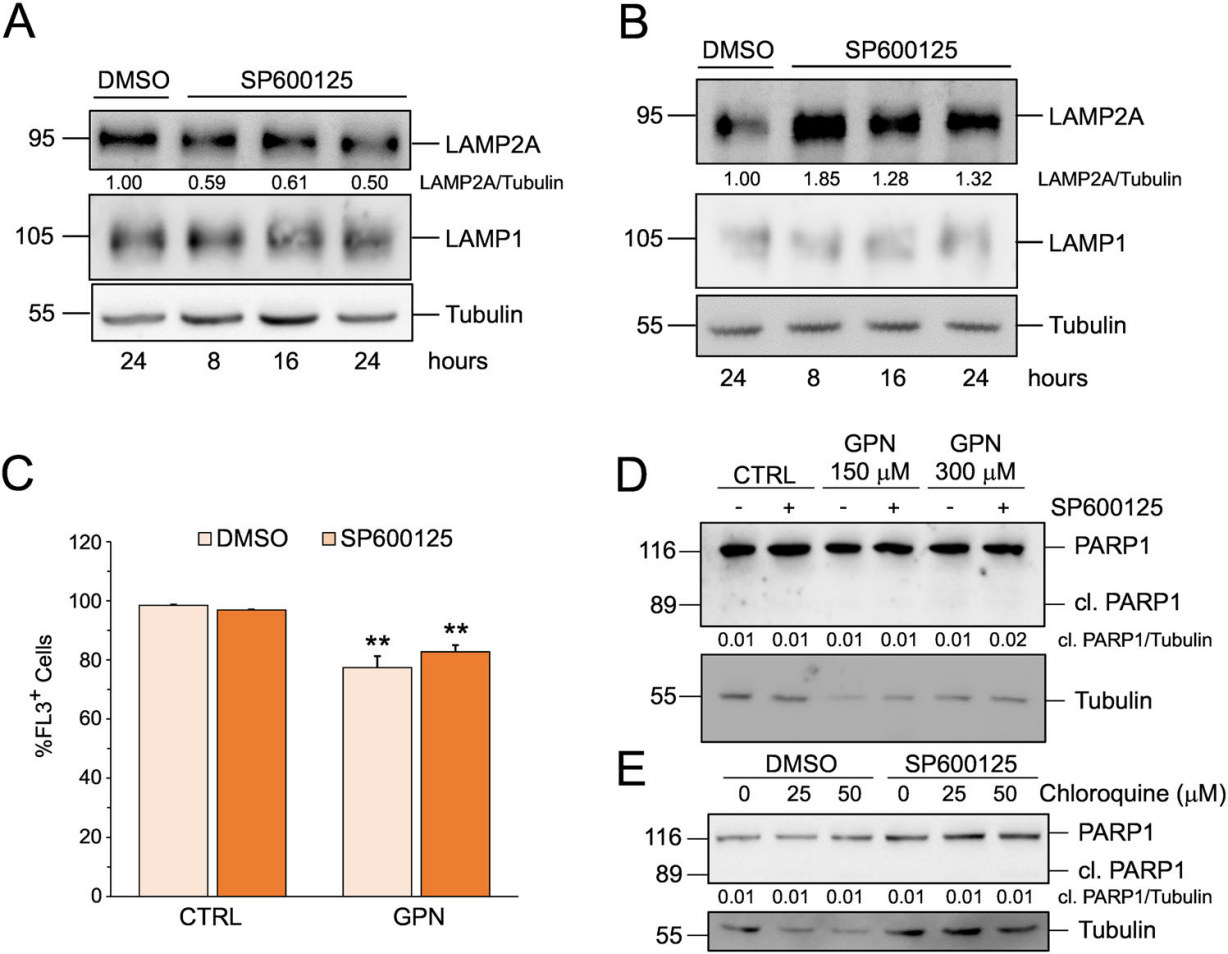
JNK regulation of LAMP2A is cancer-cell specific. **A** and **B** Western blot analyses of LAMP2A and LAMP1 expression in HepG2 (**A**) and HuS (**B**) cells treated with 10 μM SP600125 for the indicated times. **C** Cytofluorometric analysis of intracellular acidification using acridine orange in HuS cells pre-treated for 6 h with 10 μM SP600125 and then treated with 300 μM GPN for an additional 2 h. Data are expressed as mean ± SEM of *n* = 3 independent experiments. **p < 0.01 vs. CTRL. **D** and **E** Western blot analysis of PARP1 in HuS cells treated for 24 h with the indicated concentration of the lysosomotropic agents GPN (**D**) and chloroquine (**E**) in the presence or absence of 10 μM SP600125. Where applicable, tubulin was used as a loading control. Western blots are representative of *n* = 3 independent experiments showing similar results.

To verify whether the effect of JNK inhibition on LAMP2A was cell-line specific or a general phenomenon, we analyzed LAMP2A expression in another HCC cell line, HepG2, and in the immortalized hepatocyte cell line, HuS. Both cell lines were treated with 10 μM SP600125 for 8, 16, and 24 h. As shown in Fig. 3A and B, JNK inhibition caused a decrease of LAMP2A in HepG2 cells, confirming the result obtained in Hep3B, while in immortalized hepatocytes JNK inhibition caused an increase, rather than a decrease of LAMP2A. These data demonstrate that JNK regulation of LAMP2A is cancer cell-specific. Next, we asked whether the different effect of JNK inhibition in tumor and normal cells could impact the response to lysosomotropic agents. Differently from Hep3B, JNK inhibition in HuS neither exacerbated the decrease of lysosomal acidification induced by GPN (Fig. 3C), nor it increased their sensitivity to GPN or CQ (Fig. 3D and E). Altogether, these results indicated that JNK controls of LAMP2A expression and influences cell sensitivity lysosomotropic agents only in HCC cells, revealing a cancer-specific regulatory node that could be exploited for therapeutic purposes.

### JNK1 but not JNK2 regulates LAMP2A

**Fig. 4:**
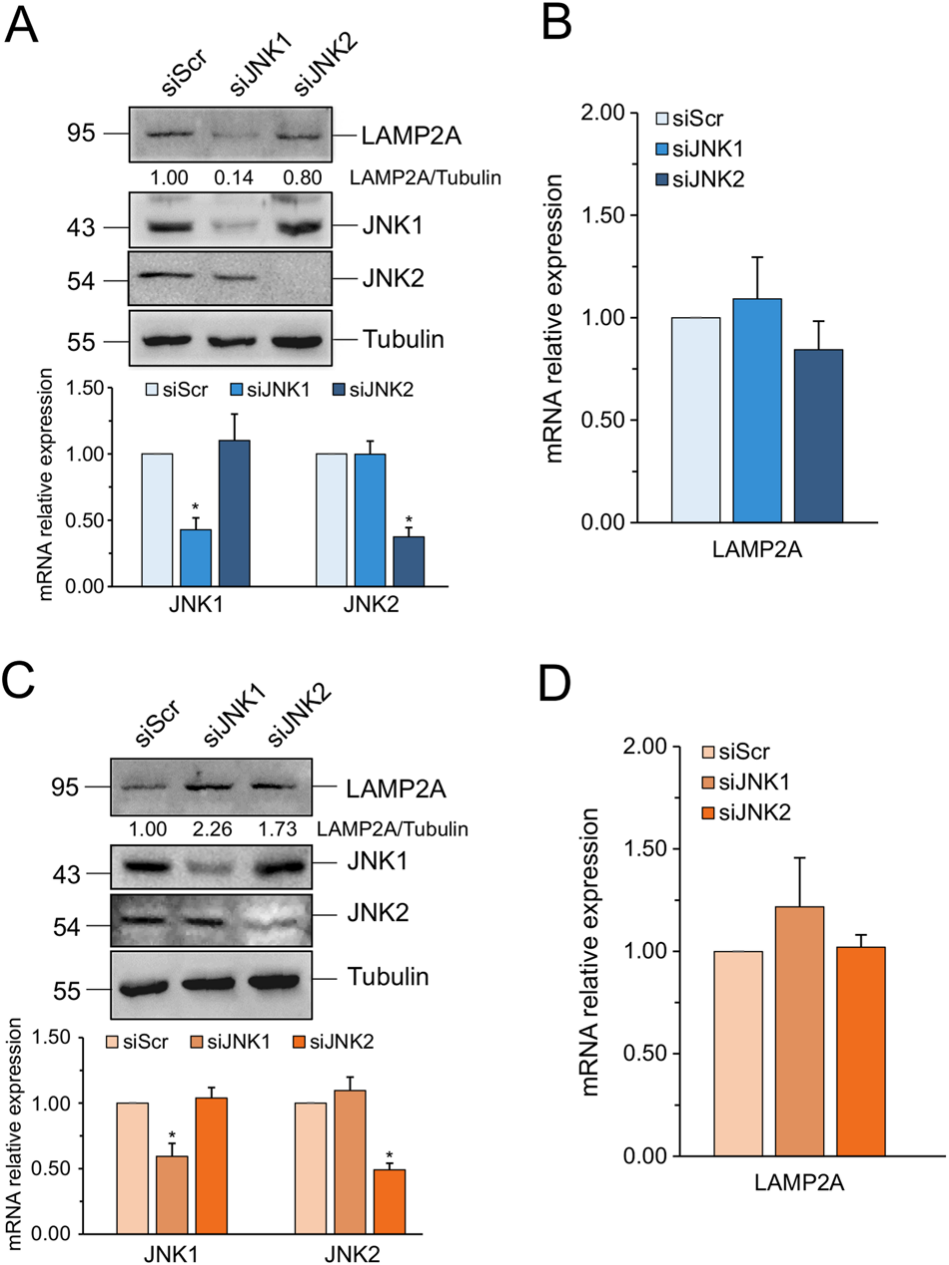
JNK1 but not JNK2 regulates LAMP2A. **A–C** (Upper panel) Western blot analyses of LAMP2A expression Hep3B (**A**) and HuS cells (**C**) transfected with siScr or siRNA specific for either JNK1 or JNK2. (Lower panel) Real-time qPCR analysis of JNK1 and JNK2 mRNA expression in Hep3B transfected with siScr or siRNA specific for either JNK1 or JNK2. Data are expressed as mean ± SEM of *n* = 3 independent experiments. *p < 0.05 vs. siScr. **B**–**D** Real-time qPCR analysis of LAMP2A mRNA expression in Hep3B and HuS cells transfected with siScr or siRNA specific for either JNK1 or JNK2. Data are expressed as mean ± SEM of *n* = 3 independent experiments. β-Actin was used as a reference gene. In Western blots, tubulin was used as a loading control, while JNK1 and JNK2 were used as control of knockdown specificity and efficacy. Western blots are representative of *n* = 3 independent experiments showing similar results.

Since three different JNK isoforms exist, we wanted to check which one regulated LAMP2A. Since JNK3 is expressed only in the brain and heart, we focused on the other two isoforms, JNK1 and JNK2. We transfected Hep3B cells with specific siRNA against JNK1 or JNK2 and analyzed LAMP2A expression by western blot and real-time qPCR 48 h later. Figure 4A shows that only JNK1 silencing decreased LAMP2A protein expression. On the contrary, the silencing of JNK2 did not have any effect. Consistent with the results obtained with the JNK inhibitor, no changes in mRNA could be detected (Fig. 4B), confirming that JNK exclusively impacted LAMP2A protein. Finally, the silencing of JNK1 and JNK2 did not cause any decrease in the expression of LAMP2A in HuS cells (Fig. 4C and D), further confirming that the regulation of LAMP2A by JNK is cancer-specific.

## Discussion

Alteration in the lysosomal compartment is an event that accompanies neoplastic transformation and tumor progression. Increase of lysosomal volume and overexpression of hydrolytic enzymes are common features of cancer cells, which strongly rely on the lysosomal function to obtain building blocks required for tumor growth^8^. Lysosomes also favors the invasive potential of tumor cells, through the extracellular release of hydrolases that degrade the extracellular matrix, and chemoresistance, by sequestering anti-cancer drugs and avoiding them to reach their targets^28^. While it is clear that lysosomal alterations favor tumor growth, at the same time they make lysosomes more fragile than normal cells and more prone to LMP, which can cause the release of proteases into the cytosol, ultimately leading to activation of apoptotic and non-apoptotic cell death mechanisms. Thus, strategies that induce LMP or make lysosomes more prone to LMP have the potential to kill cancer cells or resensitize them to chemotherapy. This strategy acquires great importance in those type of cancer, like HCC, which are highly resistant to chemotherapy and for which the therapeutic options are limited^29^. Along this line, in this paper we identify a novel role of the protein kinase JNK as a positive regulator of lysosomal homeostasis in HCC. Inhibition of JNK does not significantly influence lysosomal functionality and integrity per se, but it potentiates the cytotoxic effects of lysosomotropic agents. Indeed, co-treatment of HCC cells with the JNK inhibitor SP600125 and the lysosomotropic agent GPN strongly reduces cellular acidification, which mainly depends on the lysosomes, and promotes the appearance of galectin 3 puncta and cathepsin B in the cytosol, both indicator of lysosomal damage. As a consequence of lysosomal damage, the combination of JNK inhibitor and lysosomotropic agents causes more sustained activation of cell death, revealing a synthetic lethal interaction between the two drugs^30^. JNK is a known therapeutic target for HCC^31^, in which it is often activated and contributes to tumorigenesis and chemoresistance^32^. The regulation of lysosomal homeostasis and protection from LMP could be another way it exerts tumor-promoting functions. Mechanistically, inhibition of JNK reduces the expression of LAMP2A, the main LAMP2 isoform expressed in the liver and a key protein, together with LAMP1, for the maintenance of the integrity of the lysosomal membrane. The evidence that LAMP1 is not influenced by JNK inhibition indicates that the activity of JNK is specific for LAMP2A and do not involve the lysosome as a whole. Importantly, our data show that JNK regulation of LAMP2A occurs only in cancer cells, while in non-transformed liver cells JNK inhibition does not cause a decrease of LAMP2A expression and, in turn, does not synergize with lysosomotropic agents to promote cell death. The causes of this difference are not known yet. We can hypothesize that it may be the consequence of oncogene-driven lysosomal alterations that render LAMP2A intrinsically unstable and more susceptible to degradation, as shown by others^33^, or it could depend on the presence of redundant pathways regulating LAMP2A in normal cells that are lacking in cancer cells, making the latter more dependent on JNK. Irrespective of the underlying reason, this evidence shedding light on the existence of a cancer-specific druggable signaling pathway is extremely important as it may represent a safe way to target cancer cells with minimal side effects towards normal cells. At a deeper molecular level, JNK inhibition destabilizes LAMP2A and increase its turnover, while we could not observe any changes in mRNA levels. Unexpectedly, the decline of LAMP2A levels is prevented by inhibition of the proteasome, while the inhibition of lysosomal activity failed. Although we still do not know how JNK influences LAMP2A stability, it is highly unlikely that this occurs when LAMP2A is at the lysosomes, since lysosomal proteases regulate the turnover of lysosomal LAMP2A^34,35^, and it would be hard to envision how the proteasome could degrade it. A more likely possibility is that JNK regulates LAMP2A trafficking from the Golgi to the lysosomes or the processing of LAMP2A in the endoplasmic reticulum (ER). If LAMP2A is misfolded or incorrectly modified (e.g. glycosylated), it could be transported back from the ER to the cytosol and degraded by the proteasome, in a process known as ERAD (endoplasmic-reticulum-associated protein degradation)^36^. It is also possible that JNK directly phosphorylates LAMP2A, similarly to what has already been demonstrated for p38^18^. JNK family of kinases comprises three isoforms, among which JNK3 is expressed mostly in the heart and the brain, while the other two isoforms, JNK1 and JNK2, are broadly expressed in most tissues. Specific downregulation of the two isoforms by siRNA revealed that only JNK1 controls LAMP2A, while JNK2 seems to have no role. This result is not surprising, as isoform-specific functions of JNK family members, as well as a different role in tumorigenesis and tumor progression, have extensively been described^22^. Specifically, in the context of the liver, JNK1 is the isoform found activated in HCC and shown to promote liver cell proliferation and tumor formation^37,38^. Overall, our work uncovers a novel, cancer-specific role of JNK1 as keeper of lysosomal homeostasis in HCC cells, laying the ground for future evaluation of combination therapy with lysosomotropic agents.

## Materials and methods

### Cell culture

Hep3B, HuH7, and HepG2 cells were obtained from the European Collection of Cell Culture (ECACC) and grown in DMEM (Hep3B, HuH7) and RPM1640 (HepG2), supplemented with 10% FBS and 1% penicillin–streptomycin. HuS cells were a gift of Dr. Vinicio Carloni, Università degli Studi di Firenze and were grown in DMEM containing 4.5 g/l of glucose and supplemented with 10% FBS, 5 ng/ml EGF, 0.42 μg/ml insulin, 20 ng/ml selenium, 1% DMSO, and 1% penicillin–streptomycin. Cells have been authenticated by STR PCR by the supplier and routinely tested for Mycoplasma contamination using Myco Alert (Cambrex).

### Transfection

The following siRNAs (all from Sigma Aldrich) were used: JNK1 (5′-TTTCAACGTAAGTCCTTACTG-3′); JNK2 #2 (5′-GAATAACCTGACATAAGTTAG-3′); siRNA Universal Negative Control (SIC001) was used as negative control; all siRNAs were used at the final concentration of 25 nM and cells assayed 48 h after transfection. pcDNA3-LAMP2A plasmid was a gift of Prof. Renate Kain, Medizinische Universität Wien. All siRNAs and plasmids were transfected using Lipofectamine 3000 (Life Technologies), following the manufacture’s instruction.

### Immunoblotting

Cells were lysed in RIPA buffer (150 mM NaCl, 25 mM Tris–HCl pH 7.4, 1 mM EDTA, 1 mM Na_3_VO_4_, 10 mM NaF, and protease inhibitor cocktail) and centrifuged at 14,000 × *g* for 15 min at 4 °C. Supernatants were collected, samples electrophoresed by SDS–PAGE and blotted onto a nitrocellulose membrane (GE Healthcare, 10402495). Primary antibodies used were as follows (dilution 1:1000 unless otherwise stated): anti-LAMP2A (#ab18528) was from Abcam; anti-Cathepsin B (#31718) and anti-PARP1 (#9542) were from Cell Signaling Technology; anti-GAPDH (#sc-47724), anti-JNK1 (#sc-1648), anti-JNK2 (#sc-271133) anti-LAMP1 (#sc-20011), and anti-Tubulin (#sc-5286) were from Santa Cruz Biotechnology. Immunoblots were acquired using a Fluorchem imaging system (Alpha Innotech) and quantified using the AlphaEaseFC software (Alpha Innotech).

### Immunofluorescence

Cells seeded on coverslips were pre-treated for 6 h with 10 μM SP600126 and then treated for an additional 2 h with 300 μM GPN. Cells were then fixed for 15 min with 10% formalin solution and solubilized for 20 min with PBS–digitonin (50 μg/ml). After 30 min blocking with PBS–BSA 1%, cells were incubated with Galectin-3 antibody (sc-32790; 1:100 dilution; Santa Cruz Biotechnology) for 1 h at RT and then with an anti-mouse Alexa Fluor™ 568 (1:1000) for 1 h at RT in the dark. Nuclei were stained with Hoechst 33342 (1:2000). Images were acquired using an Axio Observer microscope (Zeiss) equipped with a Plan‐Apochromat Plan-Apochromat ×100/1.40 oil objective lens and connected to a Zeiss AxioCam. Quantification of Galectin-3 puncta were performed using the ImageJ software.

### Quantitative real-time PCR

Cells were homogenized in TRI Reagent (Sigma Aldrich) and RNA was extracted according to the manufacturer’s instructions. Total RNA was resuspended in RNase-free water and 1 µg of total RNA was used to generate cDNA using the PrimeScript RT-PCR Kit (Takara Bio). Real-time PCR was performed using the PowerUp SYBR Green Master Mix (Thermo Fisher Scientific) on a QuantStudio 3 Real-Time PCR system (Thermo Fisher Scientific). The following primer pairs were used: JNK1 (Forward 5′-TGTGTGGAATCAAGCACCTTC-3′, Reverse 5′-AGGCGTCATCATAAAACTCGTTC-3′); JNK2 (Forward 5′-GAAACTAAGCCGTCCTTTTCAGA-3′, Reverse 5′-TCCAGCTCCATGTGAATAACCT-3′); LAMP2A (Forward 5′-ACTGTTTCAGTGTCTGGAGCAT-3′, Reverse 5′-GCACTGCAGTCTTGAGCTGT-3′); β-Actin (Forward 5′-GGCCGAGGACTTTGATTGCA-3′, Reverse 5′-GGGACTTCCTGTAACAACGCA-3′). All reactions were run as triplicates. Data were analyzed by the Design and Analysis Application (Thermo Fisher Scientific) using the second derivative maximum method. The fold changes in mRNA levels were relative to the control after normalization to the internal standard β-Actin.

### Analysis of intracellular acidification

At the end of the experimental time, cells were incubated with 1 μg/ml acridine orange for 10 min at 37 °C and analyzed by FACS. The increase of FL-3 emitting cells is proportional to the increase of intracellular acidification, mostly dependent on lysosomes.

### Extraction of cytosolic proteins

Cells were detached by trypsinization and centrifuged at 400 × *g* for 5 min at 4 °C. Pellets were resuspended in digitonin lysis buffer (150 mM NaCl, 50 mM HEPES, pH 7.4, and 100 μg/ml Digitonin), incubated for 10 min at 4 °C and centrifuged at 3000 × *g* for 10 min. Supernatants containing cytosolic proteins were transferred into new clean tubes, while pellets containing the membrane-bound organelles were lysed in RIPA buffer. Samples were analyzed by SDS–PAGE as already described.

### Statistical analysis

Values are expressed as means ± SEM of at least three independent experiments; *p* values were calculated using unpaired two-tailed Student’s *t*-test. A *p*-value ≤ 0.05 was considered statistically significant.
